# Supplementary material for: Loratadine Decreases Methicillin-Resistant Staphylococcus aureus Virulence through Global Hemolysin Regulators vraSR and agr
Source: ACS Omega. 2026 Oct 5;11(41):63059–74. doi: 10.1021/acsomega.6c05740 (PMC13688506; doi:10.1021/acsomega.6c05740)
Supplement: Supplementary file 1 [file ao6c05740_si_001.pdf]

Supporting Information

Loratadine decreases methicillin-resistant *Staphylococcus aureus* virulence through global hemolysin regulators *vraSR* and *agr*.

Chase Dillon[a*], Isabel Marshall[bd*], Maggy Henkel[b], Halie Balogh[ce], Robin Stempel[cf], E. Renee Monge[b], Gabriel Z. Valenzano[cg], Meghan S. Blackledge[c#], Heather B. Miller[c#]

[a]Department of Neuroscience, High Point University, High Point, North Carolina
[b]Department of Biology, High Point University, High Point, North Carolina
[c]Department of Chemistry, High Point University, High Point, North Carolina

Present addresses:
[d]Isabel Marshall: University of Georgia, Department of Epidemiology, 101 Buck Road, Athens, Georgia 30601
[e]Halie Balogh: Wake Forest University School of Medicine, 1 Medical Center Blvd, Winston-Salem, North Carolina 27157
[f]Robin Stempel: University of Bristol, Department of Engineering Biology, Queens Road, Bristol, BS8 1QU, United Kingdom
[g]Gabriel Valenzano: Virginia Tech University, Department of Biochemistry, 340 W Campus Dr, Blacksburg, Virginia 24060

*Co-first authors: Chase Dillon and Isabel Marshall

#Correspondent footnote: Meghan S. Blackledge, mblackle@highpoint.edu, Heather B. Miller, hmiller@highpoint.edu

Table of Contents

**Supporting Tables**	**Page**
S1 - 2-way ANOVA results summarizing effects of loratadine-induced changes to hemolysis	4
S2 - 2-way ANOVA results analyzing effects of loratadine and stk1 on alpha hemolysis	5
S3 - 2-way ANOVA results analyzing effects of loratadine and stk1 on beta hemolysis	5
S4 - 2-way ANOVA results analyzing effects of loratadine and stp1 on alpha hemolysis	5
S5 - 2-way ANOVA results analyzing effects of loratadine and stp1 on beta hemolysis	6
S6 - 2-way ANOVA results summarizing effects of compound 8-induced changes to hemolysis	6
S7 - 2-way ANOVA results analyzing effects of loratadine and sarA on alpha hemolysis	7
S8 - 2-way ANOVA results analyzing effects of loratadine and sarZ on alpha hemolysis	7
S9 - 2-way ANOVA results analyzing effects of loratadine and sarA on beta hemolysis	7
S10 - 2-way ANOVA results analyzing effects of loratadine and sarZ on beta hemolysis	8
S11 - 2-way ANOVA results analyzing effects of loratadine and sarS on alpha hemolysis	8
S12 - 2-way ANOVA results analyzing effects of loratadine and sarT on alpha hemolysis	8
S13 - 2-way ANOVA results analyzing effects of loratadine and sarU on alpha hemolysis	9
S14 - 2-way ANOVA results analyzing effects of loratadine and sarV on alpha hemolysis	9
S15 - 2-way ANOVA results analyzing effects of loratadine and sarX on alpha hemolysis	9
S16 - 2-way ANOVA results analyzing effects of loratadine and sarS on beta hemolysis	10
S17 - 2-way ANOVA results analyzing effects of loratadine and sarT on beta hemolysis	10
S18 - 2-way ANOVA results analyzing effects of loratadine and sarU on beta hemolysis	10
S19 - 2-way ANOVA results analyzing effects of loratadine and sarV on beta hemolysis	11
S20 - 2-way ANOVA results analyzing effects of loratadine and sarX on beta hemolysis	11
S21 - 2-way ANOVA results analyzing effects of loratadine and saeR on alpha hemolysis	11

**Supporting Tables**	**Page**
S22 - 2-way ANOVA results analyzing effects of loratadine and saeS on alpha hemolysis	12
S23 - 2-way ANOVA results analyzing effects of loratadine and saeR on beta hemolysis	12
S24 - 2-way ANOVA results analyzing effects of loratadine and saeS on beta hemolysis	12
S25 - 2-way ANOVA results analyzing effects of loratadine and arlR on alpha hemolysis	13
S26 - 2-way ANOVA results analyzing effects of loratadine and arlS on alpha hemolysis	13
S27 - 2-way ANOVA results analyzing effects of loratadine and arlR on beta hemolysis	13
S28 - 2-way ANOVA results analyzing effects of loratadine and arlS on beta hemolysis	14
S29 - 2-way ANOVA results analyzing effects of loratadine and codY on alpha hemolysis	14
S30 - 2-way ANOVA results analyzing effects of loratadine and codY on beta hemolysis	14
S31-Complete DEG data sets for loratadine available in an Excel workbook	
S32-34 – Loratadine GO Enrichment results available in an Excel workbook	
S35-37 – Loratadine KEGG Enrichment results available in an Excel workbook	
S38 - 2-way ANOVA results analyzing effects of loratadine and time on infected EA.hy926 cell roundness	15
S39 - 2-way ANOVA results analyzing effects of loratadine and time on uninfected EA.hy926 cell roundness	15
S40 - 2-way ANOVA results analyzing effects of loratadine and time on uninfected HeLa cell roundness	15
S41- Human cell line infection model RT-qPCR results with low/no amplification	16
S42 – Additional quality control details of RNA samples	16
S43 – RNA-seq read quality control	17
S44 – Mapped reads summary	17
S45 - qPCR primer information	18

**Supporting Figures**	**Page**
S1 - Quantitative hemolysis assays analyzing effects of compound 8 and sarA and sarZ	18
S2 - Quantitative hemolysis assays analyzing effects of compound 8 and other sar genes	19
S3 - Quantitative hemolysis assays analyzing effects of compound 8 and saeR and saeS	19
S4 - Quantitative hemolysis assays analyzing effects of compound 8 and arlR and arlS	20
S5 - Quantitative hemolysis assays analyzing effects of compound 8 and codY	20
S6 – RT-qPCR validation of loratadine modulating multiple hemolysin genes in vitro	21
S7 - Temporary rounding of host cells treated with loratadine	22
S8 - Cytotoxicity of compound 8 and hemolysin mRNA levels in the human cell line infection model	23
S9 - Agarose gel electrophoresis of RT-qPCR products	24

Table S1: 2-way ANOVA results summarizing effects of loratadine-induced changes to hemolysis. The gene disruption and loratadine treatment were the column and row factors, respectively in the analysis. NS = not statistically significant in this analysis (p>0.05), NA = not applicable given that there is a significant interaction effect making column and row effects difficult to interpret. Green highlighted cells indicate mutant strains that produced statistically significant interaction effects in either alpha or beta hemolysin activity.

	**Rabbit blood (alpha hemolysin)**			**Sheep blood (beta hemolysin)**		
**Tn Mutant Strain**	**Gene Disruption Effect**	**Loratadine Effect**	**Interaction Effect**	**Gene Disruption Effect**	**Loratadine Effect**	**Interaction Effect**
Tn::stk1	NA	NA	p=0.0379	NS	p=0.0003	NS
Tn::stp1	NS	p=0.0341	NS	NS	p=0.0135	NS
Tn::sarA	NS	p=0.0547	NS	NA	NA	p=0.0072
Tn::sarZ	NS	p=0.0351	NS	NS	p=0.0118	NS
Tn::sarS	p=0.0004	p=0.0455	NS	NA	NA	p=0.0470
Tn::sarT	NS	p=0.0452	NS	NA	NA	p=0.0470
Tn::sarU	NA	NA	p=0.0060	NS	p=0.0015	NS
Tn::sarV	NS	NS	NS	NS	p=0.0066	NS
Tn::sarX	NA	NA	p=0.0037	NS	p=0.0036	NS
Tn::saeR	p<0.0001	p=0.0011	NS	NS	p=0.0009	NS
Tn::saeS	p<0.0001	p=0.0485	NS	p=0.0404	p=0.0022	NS
Tn::arlR	NS	p=0.0484	NS	NS	p=0.001	NS
Tn::arlS	NA	NA	p=0.0233	NS	p=0.003	NS
Tn::codY	NA	NA	p=0.0385	NS	p=0.001	NS

Table S2: 2-way ANOVA results analyzing effects of loratadine and stk1 on alpha hemolysis. The gene disruption and loratadine treatment were the column and row factors, respectively in the analysis. ns = not statistically significant in this analysis (p>0.05).

**Source of Variation**	% total variation	P value	P value summary	Significant?	
Interaction	15.32	0.0379	*	Yes	
Loratadine	47.21	0.0024	**	Yes	
Gene disruption of stk1	17.59	0.0288	*	Yes	
**ANOVA table**	SS	DF	MS	F (DFn, DFd)	P value
Interaction	1731	1	1731	F (1, 8) = 6.164	P=0.0379
Loratadine	5338	1	5338	F (1, 8) = 19.00	P=0.0024
Gene disruption of stk1	1989	1	1989	F (1, 8) = 7.081	P=0.0288
Residual	2247	8	280.9		

Table S3: 2-way ANOVA results analyzing effects of loratadine and stp1 on alpha hemolysis. The gene disruption and loratadine treatment were the column and row factors, respectively in the analysis. ns = not statistically significant in this analysis (p>0.05).

**Source of Variation**	% total variation	P value	P value summary	Significant?	
Interaction	11.17	0.1851	ns	No	
Loratadine	34.57	0.0341	*	Yes	
Gene disruption of stp1	11.79	0.1745	ns	No	
**ANOVA table**	SS	DF	MS	F (DFn, DFd)	P value
Interaction	129.9	1	129.9	F (1, 8) = 2.103	P=0.1851
Loratadine	402.1	1	402.1	F (1, 8) = 6.510	P=0.0341
Gene disruption of stp1	137.1	1	137.1	F (1, 8) = 2.220	P=0.1745
Residual	494.1	8	61.76		

Table S4: 2-way ANOVA results analyzing effects of loratadine and stk1 on beta hemolysis. The gene disruption and loratadine treatment were the column and row factors, respectively in the analysis. ns = not statistically significant in this analysis (p>0.05).

**Source of Variation**	% total variation	P value	P value summary	Significant?	
Interaction	1.181	0.4744	ns	No	
Loratadine	77.87	0.0003	***	Yes	
Gene disruption of stk1	4.172	0.1961	ns	No	
**ANOVA table**	SS	DF	MS	F (DFn, DFd)	P value
Interaction	44.42	1	44.42	F (1, 8) = 0.5634	P=0.4744
Loratadine	2928	1	2928	F (1, 8) = 37.14	P=0.0003
Gene disruption of stk1	156.9	1	156.9	F (1, 8) = 1.990	P=0.1961
Residual	630.8	8	78.85		

Table S5: 2-way ANOVA results analyzing effects of loratadine and stp1 on beta hemolysis. The gene disruption and loratadine treatment were the column and row factors, respectively in the analysis. ns = not statistically significant in this analysis (p>0.05).

**Source of Variation**	% total variation	P value	P value summary	Significant?	
Interaction	8.644	0.2052	ns	No	
Loratadine	45.21	0.0135	*	Yes	
Gene disruption stp1	9.781	0.1806	ns	No	
**ANOVA table**	**SS**	**DF**	**MS**	**F (DFn, DFd)**	**P value**
Interaction	341.9	1	341.9	F (1, 8) = 1.902	P=0.2052
Loratadine	1788	1	1788	F (1, 8) = 9.946	P=0.0135
Gene disruption stp1	386.9	1	386.9	F (1, 8) = 2.152	P=0.1806
Residual	1438	8	179.8		

Table S6: 2-way ANOVA summary results analyzing effects of compound 8-induced changes to hemolysis. The gene disruption and compound 8 treatment were the column and row factors, respectively in the analysis. NS = not statistically significant in this analysis (p>0.05).

	**Rabbit blood (alpha hemolysin)**			**Sheep blood (beta hemolysin)**		
**Transposon Mutant Strain**	**Gene Disruption Effect**	**Compound 8 Effect**	**Interaction Effect**	**Gene Disruption Effect**	**Compound 8 Effect**	**Interaction Effect**
Tn::stk1	p=0.0353	NS	NS	NS	NS	NS
Tn::stp1	NS	NS	NS	NS	NS	NS
Tn::sarA	p=0.0156	NS	NS	p=0.0008	NS	NS
Tn::sarZ	NS	NS	NS	NS	NS	NS
Tn::sarS	p=0.0013	NS	NS	p=0.157	NS	NS
Tn::sarT	NS	NS	NS	p=0.0145	NS	NS
Tn::sarU	p=0.0001	NS	NS	p=0.0419	NS	NS
Tn::sarV	p<0.0001	NS	NS	NS	NS	NS
Tn::sarX	p=0.0095	NS	NS	NS	NS	NS
Tn::saeR	p<0.0001	NS	NS	NS	NS	NS
Tn::saeS	p<0.0001	NS	NS	p=0.0412	NS	NS
Tn::arlR	NS	NS	NS	NS	NS	NS
Tn::arlS	p=0.0022	NS	NS	NS	NS	NS
Tn::codY	p<0.0001	NS	NS	NS	NS	NS

Table S7: 2-way ANOVA results analyzing effects of loratadine and *sarA* on alpha hemolysis. The gene disruption and loratadine treatment were the column and row factors, respectively in the analysis. ns = not statistically significant in this analysis (p>0.05).

**Source of Variation**	**% total variation**	**P value**	**P value summary**	**Significant?**	
Interaction	15.99	0.1524	ns	No	
Loratadine	32.32	0.0547	ns	No	
Gene disruption sarA	0.5486	0.7770	ns	No	
**ANOVA table**	**SS**	**DF**	**MS**	**F (DFn, DFd)**	**P value**
Interaction	168.6	1	168.6	F (1, 8) = 2.501	P=0.1524
Loratadine	340.8	1	340.8	F (1, 8) = 5.055	P=0.0547
Gene disruption sarA	5.786	1	5.786	F (1, 8) = 0.08581	P=0.7770
Residual	539.4	8	67.42		

Table S8: 2-way ANOVA results analyzing effects of loratadine and *sarZ* on alpha hemolysis. The gene disruption and loratadine treatment were the column and row factors, respectively in the analysis. ns = not statistically significant in this analysis (p>0.05).

**Source of Variation**	**% total variation**	**P value**	**P value summary**	**Significant**	
Interaction	0.3719	0.8221	ns	No	
Loratadine	44.19	0.0351	*	Yes	
Gene disruption	0.3484	0.8277	ns	No	
**ANOVA table**	**SS**	**DF**	**MS**	**F (DFn,**	**P value**
Interaction	6.983	1	6.983	F (1, 8) =	P=0.8221
Loratadine	829.7	1	829.7	F (1, 8) =	P=0.0351
Gene disruption	6.542	1	6.542	F (1, 8) =	P=0.8277
Residual	1034	8	129.3		

Table S9: 2-way ANOVA results analyzing effects of loratadine and *sarA* on beta hemolysis. The gene disruption and loratadine treatment were the column and row factors, respectively in the analysis. ns = not statistically significant in this analysis (p>0.05).

**Source of Variation**	**% total variation**	**P value**	**P value summary**	**Significant?**	
Interaction	23.74	0.0072	**	Yes	
Loratadine	21.39	0.0094	**	Yes	
Gene disruption	40.03	0.0017	**	Yes	
**ANOVA table**	**SS**	**DF**	**MS**	**F (DFn, DFd)**	**P value**
Interaction	972.1	1	972.1	F (1, 8) = 12.79	P=0.0072
Loratadine	876.0	1	876.0	F (1, 8) = 11.53	P=0.0094
Gene disruption	1639	1	1639	F (1, 8) = 21.57	P=0.0017
Residual	608.1	8	76.01		

Table S10: 2-way ANOVA results analyzing effects of loratadine and *sarZ* on beta hemolysis. The gene disruption and loratadine treatment were the column and row factors, respectively in the analysis. ns = not statistically significant in this analysis (p>0.05).

**Source of Variation**	**% total variation**	**P value**	**P value summary**	**Significant?**	
Interaction	3.245	0.4524	ns	No	
Loratadine	54.70	0.0118	*	Yes	
Gene disruption	0.4360	0.7796	ns	No	
**ANOVA table**	**SS**	**DF**	**MS**	**F (DFn, DFd)**	**P value**
Interaction	141.7	1	141.7	F (1, 8) = 0.6237	P=0.4524
Loratadine	2389	1	2389	F (1, 8) = 10.51	P=0.0118
Gene disruption	19.04	1	19.04	F (1, 8) = 0.08382	P=0.7796
Residual	1817	8	227.2		

Table S11: 2-way ANOVA results analyzing effects of loratadine and *sarS* on alpha hemolysis. The gene disruption and loratadine treatment were the column and row factors, respectively in the analysis. ns = not statistically significant in this analysis (p>0.05).

**Source of Variation**	**% total variation**	**P value**	**P value summary**	**Significant?**	
Interaction	1.469	0.4212	ns	No	
Loratadine	11.45	0.0455	*	Yes	
Gene disruption	70.73	0.0004	***	Yes	
**ANOVA table**	**SS**	**DF**	**MS**	**F (DFn, DFd)**	**P value**
Interaction	308.0	1	308.0	F (1, 8) = 0.7187	P=0.4212
Loratadine	2401	1	2401	F (1, 8) = 5.602	P=0.0455
Gene disruption	14827	1	14827	F (1, 8) = 34.60	P=0.0004
Residual	3428	8	428.5		

Table S12: 2-way ANOVA results analyzing effects of loratadine and *sarT* on alpha hemolysis. The gene disruption and loratadine treatment were the column and row factors, respectively in the analysis. ns = not statistically significant in this analysis (p>0.05).

**Source of Variation**	**% total variation**	**P value**	**P value summary**	**Significant?**	
Interaction	0.0001845	0.9962	ns	No	
Loratadine	45.25	0.0452	*	Yes	
Gene disruption sarT	0.02205	0.9587	ns	No	
**ANOVA table**	**SS**	**DF**	**MS**	**F (DFn, DFd)**	**P value**
Interaction	0.003598	1	0.003598	F (1, 7) = 2.413e-005	P=0.9962
Loratadine	882.6	1	882.6	F (1, 7) = 5.920	P=0.0452
Gene disruption sarT	0.4300	1	0.4300	F (1, 7) = 0.002884	P=0.9587
Residual	1044	7	149.1		

Table S13: 2-way ANOVA results analyzing effects of loratadine and *sarU* on alpha hemolysis. The gene disruption and loratadine treatment were the column and row factors, respectively in the analysis. ns = not statistically significant in this analysis (p>0.05).

**Source of Variation**	**% total variation**	**P value**	**P value summary**	**Significant?**	
Interaction	10.66	0.0060	**	Yes	
Loratadine	31.40	0.0002	***	Yes	
Gene disruption	51.74	<0.0001	****	Yes	
**ANOVA table**	**SS**	**DF**	**MS**	**F (DFn, DFd)**	**P value**
Interaction	1928	1	1928	F (1, 8) = 13.75	P=0.0060
Loratadine	5679	1	5679	F (1, 8) = 40.50	P=0.0002
Gene disruption	9360	1	9360	F (1, 8) = 66.74	P<0.0001
Residual	1122	8	140.2		

Table S14: 2-way ANOVA results analyzing effects of loratadine and *sarV* on alpha hemolysis. The gene disruption and loratadine treatment were the column and row factors, respectively in the analysis. ns = not statistically significant in this analysis (p>0.05).

**Source of Variation**	**% total variation**	**P value**	**P value**	**Significant?**	
Interaction	1.641	0.6638	ns	No	
Loratadine	33.67	0.0752	ns	No	
Gene disruption	0.2302	0.8700	ns	No	
**ANOVA table**	**SS**	**DF**	**MS**	**F (DFn, DFd)**	**P value**
Interaction	79.37	1	79.37	F (1, 8) = 0.2037	P=0.6638
Loratadine	1629	1	1629	F (1, 8) = 4.179	P=0.0752
Gene disruption	11.13	1	11.13	F (1, 8) = 0.02857	P=0.8700
Residual	3118	8	389.7		

Table S15: 2-way ANOVA results analyzing effects of loratadine and *sarX* on alpha hemolysis. The gene disruption and loratadine treatment were the column and row factors, respectively in the analysis. ns = not statistically significant in this analysis (p>0.05).

**Source of Variation**	**% total variation**	**P value**	**P value**	**Significant?**	
Interaction	13.85	0.0037	**	Yes	
Loratadine	36.73	0.0002	***	Yes	
Gene disruption	42.67	0.0001	***	Yes	
**ANOVA table**	**SS**	**DF**	**MS**	**F (DFn, DFd)**	**P value**
Interaction	2502	1	2502	F (1, 8) = 16.41	P=0.0037
Loratadine	6637	1	6637	F (1, 8) = 43.52	P=0.0002
Gene disruption	7710	1	7710	F (1, 8) = 50.56	P=0.0001
Residual	1220	8	152.5		

Table S16: 2-way ANOVA results analyzing effects of loratadine and *sarS* on beta hemolysis. The gene disruption and loratadine treatment were the column and row factors, respectively in the analysis. ns = not statistically significant in this analysis (p>0.05).

**Source of Variation**	**% total variation**	**P value**	**P value**	**Significant?**	
Interaction	20.59	0.0313	*	Yes	
Loratadine	36.98	0.0082	**	Yes	
Gene disruption	18.18	0.0400	*	Yes	
**ANOVA table**	**SS**	**DF**	**MS**	**F (DFn, DFd)**	**P value**
Interaction	674.5	1	674.5	F (1, 8) = 6.793	P=0.0313
Loratadine	1211	1	1211	F (1, 8) = 12.20	P=0.0082
Gene disruption	595.4	1	595.4	F (1, 8) = 5.997	P=0.0400
Residual	794.3	8	99.29		

Table S17: 2-way ANOVA results analyzing effects of loratadine and *sarT* on beta hemolysis. The gene disruption and loratadine treatment were the column and row factors, respectively in the analysis. ns = not statistically significant in this analysis (p>0.05).

**Source of Variation**	**% total variation**	**P value**	**P value summary**	**Significant?**	
Interaction	17.22	0.0470	*	Yes	
Loratadine	41.20	0.0067	**	Yes	
Gene disruption	16.56	0.0504	ns	No	
**ANOVA table**	**SS**	**DF**	**MS**	**F (DFn, DFd)**	**P value**
Interaction	569.4	1	569.4	F (1, 8) = 5.503	P=0.0470
Loratadine	1363	1	1363	F (1, 8) = 13.17	P=0.0067
Gene disruption	547.7	1	547.7	F (1, 8) = 5.294	P=0.0504
Residual	827.7	8	103.5		

Table S18: 2-way ANOVA results analyzing effects of loratadine and *sarU* on beta hemolysis. The gene disruption and loratadine treatment were the column and row factors, respectively in the analysis. ns = not statistically significant in this analysis (p>0.05).

**Source of Variation**	**% total variation**	**P value**	**P value**	**Significant?**	
Interaction	8.184	0.1160	ns	No	
Loratadine	58.65	0.0015	**	Yes	
Gene disruption	12.09	0.0645	ns	No	
**ANOVA table**	**SS**	**DF**	**MS**	**F (DFn, DFd)**	**P value**
Interaction	273.2	1	273.2	F (1, 8) = 3.107	P=0.1160
Loratadine	1958	1	1958	F (1, 8) = 22.27	P=0.0015
Gene disruption	403.6	1	403.6	F (1, 8) = 4.590	P=0.0645
Residual	703.5	8	87.93		

Table S19: 2-way ANOVA results analyzing effects of loratadine and *sarV* on beta hemolysis. The gene disruption and loratadine treatment were the column and row factors, respectively in the analysis. ns = not statistically significant in this analysis (p>0.05).

**Source of Variation**	**% total variation**	**P value**	**P value**	**Significant?**	
Interaction	16.99	0.0633	ns	No	
Loratadine	48.37	0.0066	**	Yes	
Gene disruption	5.358	0.2608	ns	No	
**ANOVA table**	**SS**	**DF**	**MS**	**F (DFn, DFd)**	**P value**
Interaction	511.6	1	511.6	F (1, 8) = 4.644	P=0.0633
Loratadine	1456	1	1456	F (1, 8) = 13.22	P=0.0066
Gene disruption	161.3	1	161.3	F (1, 8) = 1.464	P=0.2608
Residual	881.4	8	110.2		

Table S20: 2-way ANOVA results analyzing effects of loratadine and *sarX* on beta hemolysis. The gene disruption and loratadine treatment were the column and row factors, respectively in the analysis. ns = not statistically significant in this analysis (p>0.05).

**Source of Variation**	**% total variation**	**P value**	**P value**	**Significant?**	
Interaction	1.499	0.5539	ns	No	
Loratadine	64.76	0.0036	**	Yes	
Gene disruption	2.308	0.4655	ns	No	
**ANOVA table**	**SS**	**DF**	**MS**	**F (DFn, DFd)**	**P value**
Interaction	64.41	1	64.41	F (1, 8) = 0.3816	P=0.5539
Loratadine	2783	1	2783	F (1, 8) = 16.48	P=0.0036
Gene disruption	99.15	1	99.15	F (1, 8) = 0.5873	P=0.4655
Residual	1350	8	168.8		

Table S21: 2-way ANOVA results analyzing effects of loratadine and *saeR* on alpha hemolysis. The gene disruption and loratadine treatment were the column and row factors, respectively in the analysis. ns = not statistically significant in this analysis (p>0.05).

**Source of Variation**	**% total variation**	**P value**	**P value**	**Significant?**	
Interaction	0.5033	0.2726	ns	No	
Loratadine	8.986	0.0011	**	Yes	
Gene disruption	87.61	<0.0001	****	Yes	
**ANOVA table**	**SS**	**DF**	**MS**	**F (DFn, DFd)**	**P value**
Interaction	95.05	1	95.05	F (1, 8) = 1.388	P=0.2726
Loratadine	1697	1	1697	F (1, 8) = 24.78	P=0.0011
Gene disruption	16547	1	16547	F (1, 8) = 241.6	P<0.0001
Residual	547.9	8	68.49		

Table S22: 2-way ANOVA results analyzing effects of loratadine and *saeS* on alpha hemolysis. The gene disruption and loratadine treatment were the column and row factors, respectively in the analysis. ns = not statistically significant in this analysis (p>0.05).

Source of Variation	% total variation	P value	P value	Significant?	
Interaction	0.7208	0.0956	ns	No	
Loratadine	1.093	0.0485	*	Yes	
Gene disruption	96.57	<0.0001	****	Yes	
**ANOVA table**	**SS**	**DF**	**MS**	**F (DFn, DFd)**	**P value**
Interaction	198.6	1	198.6	F (1, 8) = 3.568	P=0.0956
Loratadine	301.2	1	301.2	F (1, 8) = 5.410	P=0.0485
Gene disruption	26610	1	26610	F (1, 8) = 478.0	P<0.0001
Residual	445.3	8	55.67		

Table S23: 2-way ANOVA results analyzing effects of loratadine and *saeR* on beta hemolysis. The gene disruption and loratadine treatment were the column and row factors, respectively in the analysis. ns = not statistically significant in this analysis (p>0.05).

Source of Variation	% total variation	P value	P value	Significant?	
Interaction	6.752	0.1407	ns	No	
Loratadine	66.22	0.0009	***	Yes	
Gene disruption	6.822	0.1389	ns	No	
**ANOVA table**	**SS**	**DF**	**MS**	**F (DFn, DFd)**	**P value**
Interaction	216.4	1	216.4	F (1, 8) = 2.673	P=0.1407
Loratadine	2122	1	2122	F (1, 8) = 26.22	P=0.0009
Gene disruption	218.6	1	218.6	F (1, 8) = 2.701	P=0.1389
Residual	647.6	8	80.95		

Table S24: 2-way ANOVA results analyzing effects of loratadine and *saeS* on beta hemolysis. The gene disruption and loratadine treatment were the column and row factors, respectively in the analysis. ns = not statistically significant in this analysis (p>0.05).

Source of Variation	% total variation	P value	P value summary	Significant?	
Interaction	13.53	0.0510	ns	No	
Loratadine	50.53	0.0022	**	Yes	
Gene disruption	15.35	0.0404	*	Yes	
**ANOVA table**	**SS**	**DF**	**MS**	**F (DFn, DFd)**	**P value**
Interaction	429.6	1	429.6	F (1, 8) = 5.259	P=0.0510
Loratadine	1604	1	1604	F (1, 8) = 19.63	P=0.0022
Gene disruption	487.3	1	487.3	F (1, 8) = 5.965	P=0.0404
Residual	653.5	8	81.69		

Table S25: 2-way ANOVA results analyzing effects of loratadine and *arlR* on alpha hemolysis. The gene disruption and loratadine treatment were the column and row factors, respectively in the analysis. ns = not statistically significant in this analysis (p>0.05).

**Source of Variation**	**% total variation**	**P value**	**P value**	**Significant?**	
Interaction	2.871	0.5385	ns	No	
Loratadine	37.63	0.0484	*	Yes	
Gene disruption	3.867	0.4772	ns	No	
**ANOVA table**	**SS**	**DF**	**MS**	**F (DFn, DFd)**	**P value**
Interaction	46.32	1	46.32	F (1, 8) = 0.4129	P=0.5385
Loratadine	607.2	1	607.2	F (1, 8) = 5.412	P=0.0484
Gene disruption	62.39	1	62.39	F (1, 8) = 0.5561	P=0.4772
Residual	897.5	8	112.2		

Table S26: 2-way ANOVA results analyzing effects of loratadine and *arlS* on alpha hemolysis. The gene disruption and loratadine treatment were the column and row factors, respectively in the analysis. ns = not statistically significant in this analysis (p>0.05).

**Source of Variation**	**% total variation**	**P value**	**P value**	**Significant?**	
Interaction	10.62	0.0233	*	Yes	
Loratadine	41.17	0.0006	***	Yes	
Gene disruption	37.35	0.0008	***	Yes	
**ANOVA table**	**SS**	**DF**	**MS**	**F (DFn, DFd)**	**P value**
Interaction	1053	1	1053	F (1, 8) = 7.828	P=0.0233
Loratadine	4084	1	4084	F (1, 8) = 30.34	P=0.0006
Gene disruption	3705	1	3705	F (1, 8) = 27.53	P=0.0008
Residual	1077	8	134.6		

Table S27: 2-way ANOVA results analyzing effects of loratadine and *arlR* on beta hemolysis. The gene disruption and loratadine treatment were the column and row factors, respectively in the analysis. ns = not statistically significant in this analysis (p>0.05).

**Source of Variation**	**% total variation**	**P value**	**P value**	**Significant?**	
Interaction	4.046	0.2683	ns	No	
Loratadine	71.66	0.0010	**	Yes	
Gene disruption	1.422	0.5007	ns	No	
**ANOVA table**	**SS**	**DF**	**MS**	**F (DFn, DFd)**	**P value**
Interaction	136.2	1	136.2	F (1, 8) = 1.415	P=0.2683
Loratadine	2411	1	2411	F (1, 8) = 25.06	P=0.0010
Gene disruption	47.84	1	47.84	F (1, 8) = 0.4972	P=0.5007
Residual	769.8	8	96.22		

Table S28: 2-way ANOVA results analyzing effects of loratadine and *arlS* on beta hemolysis. The gene disruption and loratadine treatment were the column and row factors, respectively in the analysis. ns = not statistically significant in this analysis (p>0.05).

Source of Variation	% total variation	P value	P value	Significant?	
Interaction	0.1525	0.8032	ns	No	
Loratadine	81.42	0.0003	***	Yes	
Gene disruption	0.04945	0.8870	ns	No	
**ANOVA table**	**SS**	**DF**	**MS**	**F (DFn, DFd)**	**P value**
Interaction	6.356	1	6.356	F (1, 8) = 0.06636	P=0.8032
Loratadine	3394	1	3394	F (1, 8) = 35.43	P=0.0003
Gene disruption	2.061	1	2.061	F (1, 8) = 0.02152	P=0.8870
Residual	766.3	8	95.78		

Table S29: 2-way ANOVA results analyzing effects of loratadine and *codY* on alpha hemolysis. The gene disruption and loratadine treatment were the column and row factors, respectively in the analysis. ns = not statistically significant in this analysis (p>0.05).

Source of Variation	% total variation	P value	P value	Significant?	
Interaction	7.728	0.0385	*	Yes	
Loratadine	33.57	0.0009	***	Yes	
Gene disruption	48.59	0.0003	***	Yes	
**ANOVA table**	**SS**	**DF**	**MS**	**F (DFn, DFd)**	**P value**
Interaction	841.1	1	841.1	F (1, 8) = 6.119	P=0.0385
Loratadine	3654	1	3654	F (1, 8) = 26.58	P=0.0009
Gene disruption	5289	1	5289	F (1, 8) = 38.48	P=0.0003
Residual	1100	8	137.5		

Table S30: 2-way ANOVA results analyzing effects of loratadine and *codY* on beta hemolysis. The gene disruption and loratadine treatment were the column and row factors, respectively in the analysis. ns = not statistically significant in this analysis (p>0.05).

Source of Variation	% total variation	P value	P value	Significant?	
Interaction	1.525	0.4973	ns	No	
Loratadine	74.19	0.0011	**	Yes	
Gene disruption	0.1444	0.8323	ns	No	
**ANOVA table**	**SS**	**DF**	**MS**	**F (DFn, DFd)**	**P value**
Interaction	58.09	1	58.09	F (1, 8) = 0.5055	P=0.4973
Loratadine	2825	1	2825	F (1, 8) = 24.59	P=0.0011
Gene disruption	5.499	1	5.499	F (1, 8) = 0.04785	P=0.8323
Residual	919.3	8	114.9		

Table S38: 2-way ANOVA results analyzing effects of loratadine and time on infected EA.hy926 cell roundness. The time and loratadine treatment were the column and row factors, respectively in the analysis. ns = not statistically significant in this analysis (p>0.05).

**Source of Variation**	**% total variation**	**P value**	**P value**	**Significant?**	
Interaction	10.03	0.0350	*	Yes	
Time	4.932	0.0748	ns	No	
Drug Treatment	10.42	0.0050	**	Yes	
**ANOVA table**	**SS**	**DF**	**MS**	**F (DFn, DFd)**	**P value**
Interaction	0.4571	4	0.1143	F (4, 81) = 2.72	P=0.0350
Time	0.2247	2	0.1124	F (2, 81) = 2.67	P=0.0748
Drug Treatment	0.4747	2	0.2374	F (2, 81) = 5.65	P=0.0050
Residual	3.400	81	0.04197		

Table S39: 2-way ANOVA results analyzing effects of loratadine and time on uninfected EA.hy926 cell roundness. The time and loratadine treatment were the column and row factors, respectively in the analysis. ns = not statistically significant in this analysis (p>0.05).

**Source of Variation**	**% total variation**	**P value**	**P value summary**	**Significant?**	
Interaction	3.127	0.3487	ns	No	
Time	26.67	<0.0001	****	Yes	
Drug Treatment	14.11	0.0001	***	Yes	
**ANOVA table**	**SS**	**DF**	**MS**	**F (DFn, DFd)**	**P value**
Interaction	0.1379	4	0.03448	F (4, 80) = 1.129	P=0.3487
Time	1.176	2	0.5881	F (2, 80) = 19.26	P<0.0001
Drug Treatment	0.6223	2	0.3112	F (2, 80) = 10.19	P=0.0001
Residual	2.443	80	0.03054		

Table S40: 2-way ANOVA results analyzing effects of loratadine and time on uninfected HeLa cell roundness. The time and loratadine treatment were the column and row factors, respectively in the analysis. ns = not statistically significant in this analysis (p>0.05).

**Source of Variation**	**% total variation**	**P value**	**P value summary**	**Significant?**	
Interaction	11.45	0.0088	**	Yes	
Time	7.983	0.0084	**	Yes	
Drug Treatment	16.91	<0.0001	****	Yes	
**ANOVA table**	**SS**	**DF**	**MS**	**F (DFn, DFd)**	**P value**
Interaction	0.3160	4	0.07901	F (4, 81) = 3.644	P=0.0088
Time	0.2203	2	0.1101	F (2, 81) = 5.079	P=0.0084
Drug Treatment	0.4665	2	0.2332	F (2, 81) = 10.76	P<0.0001
Residual	1.756	81	0.02168		

Table S41: Human cell line infection model RT-qPCR results with low/no amplification. Data shown are from a representative experiment with mean Ct values from duplicate reactions using untreated but infected cells. +RT = PCR reaction with cDNA as template, -RT = control PCR reaction that lacked reverse transcriptase in the cDNA synthesis step, NTC = no template control PCR reaction that lacked template and had only water and PCR mastermix. Red Ct values indicate that the Ct obtained in the +RT reaction was less than 5 Cts different from the –RT and NTC reactions. This indicates amplification of these genes is similar to background levels.

**Gene**	**WT JE2 infection**	**Tn::stk1 infection**	**Tn::stp1 infection**	**Tn::sarA infection**
	**Mean Ct**	**Meant Ct**	**Meant Ct**	**Meant Ct**
**+RT**				
16S	11.78793	25.29228	17.7547836	10.17088
hla	22.1402	32.09037	26.07072	20.2918625
hlgA	24.94802	31.7953	27.7055206	26.54224
hlgC	27.6679	28.31001	31.5830002	25.31952
hld/RNAIII	22.6522751	32.05889	27.99364	24.21673
**-RT**				
16S	30.59434	33.7104	34.16547	32.8718033
hla	28.657629	28.19838	27.77948	26.91212
hlgA	27.49573	32.69831	29.6728287	31.15741
hlgC	29.7959023	30.72838	32.3512535	25.31952
hld/RNAIII	27.39732	31.90166	32.8868256	34.2149353
**NTC**				
16S	29.67368	32.40137	34.86722	33.87514
hla	24.0518951	33.60343	27.81045	27.4429951
hlgA	28.35803	33.59329	30.29202	31.65088
hlgC	28.83661	31.24105	29.8993	31.47275
hld/RNAIII	31.9273	33.37765	31.96478	34.17091

Table S42: Additional quality control details of RNA samples. Un represents untreated sample, lor represents loratadine treated sample. Each biological replicate is labelled A, B, or C. RIN = RNA integrity number

**Sample Name**	**Concentration (ng/µL)**	**RIN**
UnA	64.8	6.4
UnB	279.2	7.8
UnC	202.0	7.7
LorA	43.2	5.8
LorB	171.2	7.1
LorC	76.4	7.7

Table S43: RNA-seq reads quality control summary. Un represents untreated sample, lor represents loratadine treated sample. Each biological replicate is labelled A, B, or C. Q20 and Q30 were calculated as the base number of Phred value > 20 or 30, respectively, divided by the total base value x 100%.

Sample name	Raw reads	Clean reads	Raw bases	Clean bases	Error rate	Q20	Q30	GC content
UnA	24119548	22181216	3.62G	3.33G	0.03	97.37	92.5	34.63
LorA	22706338	20878286	3.41G	3.14G	0.03	97.02	92.00	33.91
UnB	27424072	26201028	4.12G	3.94G	0.03	97.23	92.16	34.49
LorB	26122322	25020814	3.92G	3.76G	0.03	97.23	92.18	34.33
UnC	26598234	25372060	3.99G	3.81G	0.03	97.29	92.33	34.61
LorC	26059026	24610654	3.91G	3.7G	0.03	97.31	92.32	34.42

Table S44: Mapped reads summary. Un represents untreated sample, lor represents loratadine treated sample. Each biological replicate is labelled A, B, or C.

Sample name	UnA	UnB	UnC	LorA	LorB	LorC
**Total reads**	22181216	26201028	25372060	20878286	25020814	24610654
**Total mapped**	21817247 (98.36%)	25740613 (98.24%)	24961910 (98.38%)	19796912 (94.82%)	24449618 (97.72%)	24117202 (97.99%)
**Multiple mapped**	428988 (1.93%)	527355 (2.01%)	481473 (1.9%)	433291 (2.08%)	495271 (1.98%)	479623 (1.95%)
**Uniquely mapped**	21388259 (96.43%)	25213258 (96.23%)	24480437 (96.49%)	19363621 (92.75%)	23954347 (95.74%)	23637579 (96.05%)
**Read-1**	10701153 (48.24%)	12618727 (48.16%)	12252152 (48.29%)	9688575 (46.41%)	11988990 (47.92%)	11827116 (48.06%)
**Read-2**	10687106 (48.18%)	12594531 (48.07%)	12228285 (48.2%)	9675046 (46.34%)	11965357 (47.82%)	11810463 (47.99%)
**Reads map to '+'**	10693846 (48.21%)	12605858 (48.11%)	12239256 (48.24%)	9681823 (46.37%)	11976675 (47.87%)	11817990 (48.02%)
**Reads map to '-'**	10694413 (48.21%)	12607400 (48.12%)	12241181 (48.25%)	9681798 (46.37%)	11977672 (47.87%)	11819589 (48.03%)
**Reads mapped in proper pairs**	20116998 (90.69%)	23937836 (91.36%)	23052036 (90.86%)	18264546 (87.48%)	22627440 (90.43%)	22435994 (91.16%)
**Proper-paired reads map to different chrom**	0 (0%)	0 (0%)	0 (0%)	0 (0%)	0 (0%)	0 (0%)

Table S45: RT-qPCR primer information

Gene	Forward	Reverse	Calculated Efficiency	Reference
16S	CTGTGCACATCTTGACGGTA	TCAGCGTCAGTTACAGACCA	81%	Yarwood et al.(1)
hla	ATGAATCCTGTCGCTAATGCCG	TGACCAGCAATGGTACCTTTCG	114.04%	Balogh et al. (2)
hlgA	AGCAGTTGGTTTAATCGCCCCTTT	TTGATGATTTCTGCACCTTGGCCG	83.86%	Viering et al.(3)
hlgC	GGTGGTAATTTCCAATCAGCC	GAATGAATTCGCTTTGACGCCC	109.18%	Balogh et al. (2)
hld/RNAIII	TAATTAAGGAAGGAGTGATTTCAATG	TTTTTAGTGAATTTGTTCACTGTGTC	118.36%	Zheng et al.(4)
agrA	GTGAATTCGTAAGCATGACCCAGTTG	TGTAAGCGTGTATGTGCAGTTTCTAAA	92.80%	This work

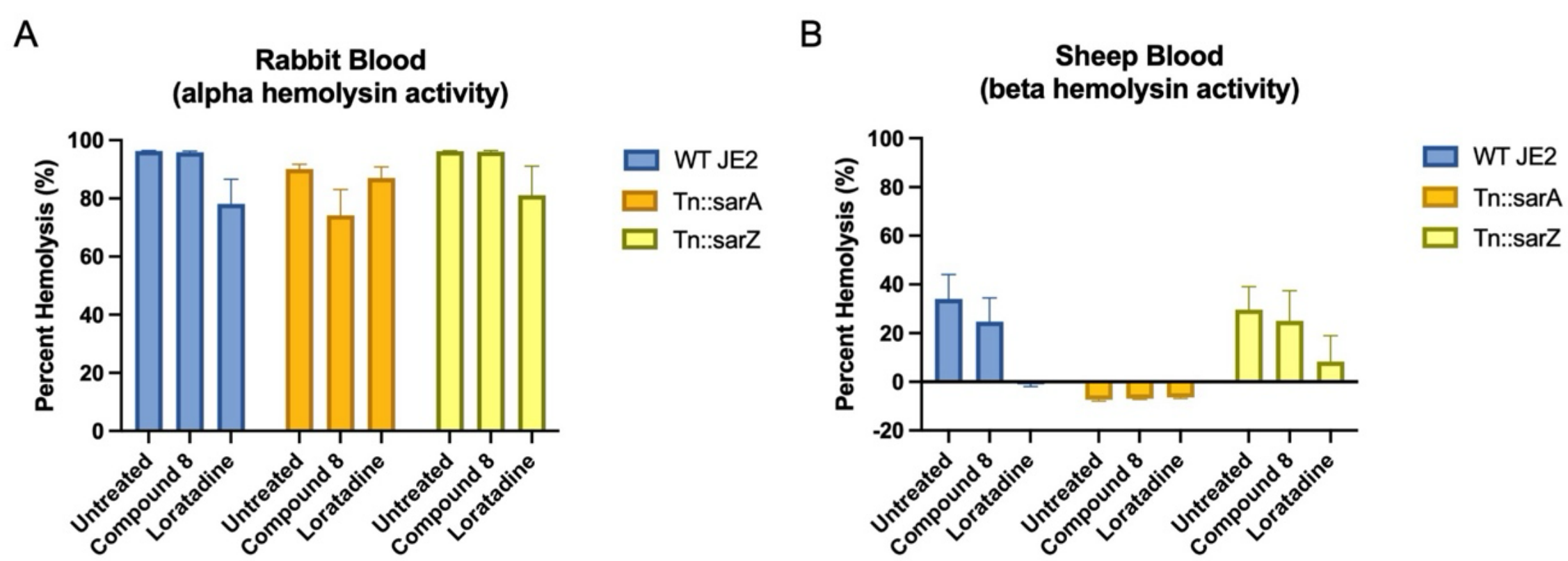

Figure S1: Quantitative hemolysis assays using (A) rabbit or (B) sheep blood reveal SarA impacting loratadine-induced reduction of beta hemolytic activity in USA300 strain JE2. Compound 8 results are also included. Mean percent hemolysis is displayed from three biological replicates. Error bars represent the standard error of the mean. Two-way ANOVAs were used to assess variance between means of every sample. Details of this analysis are shared in Tables S1 for loratadine and S6 for compound 8.

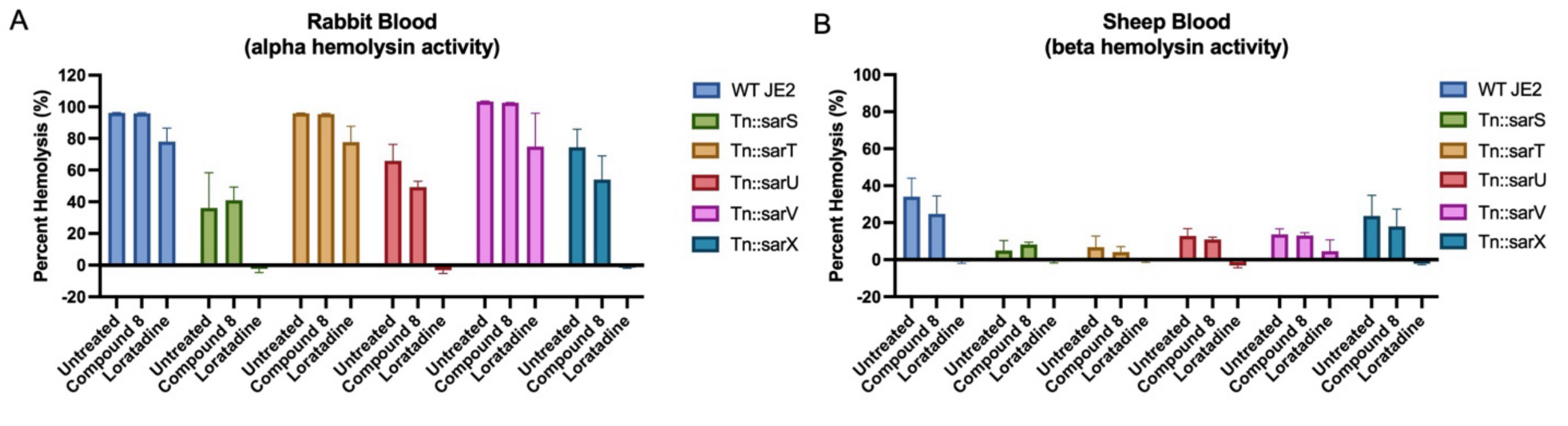

Figure S2: Quantitative hemolysis assays using (A) rabbit or (B) sheep blood reveal SarU and SarX inhibiting loratadine's reduction of alpha hemolytic activity in USA300 strain JE2. SarS and SarT impact loratadine's reduction of beta hemolytic activity. Compound 8 results are also included. In both panels, mean percent hemolysis is displayed from three biological replicates. Error bars represent the standard error of the mean. Two-way ANOVAs were used to assess variance between means of every sample. Details of this analysis are shared in Tables S1 for loratadine and S6 for compound 8.

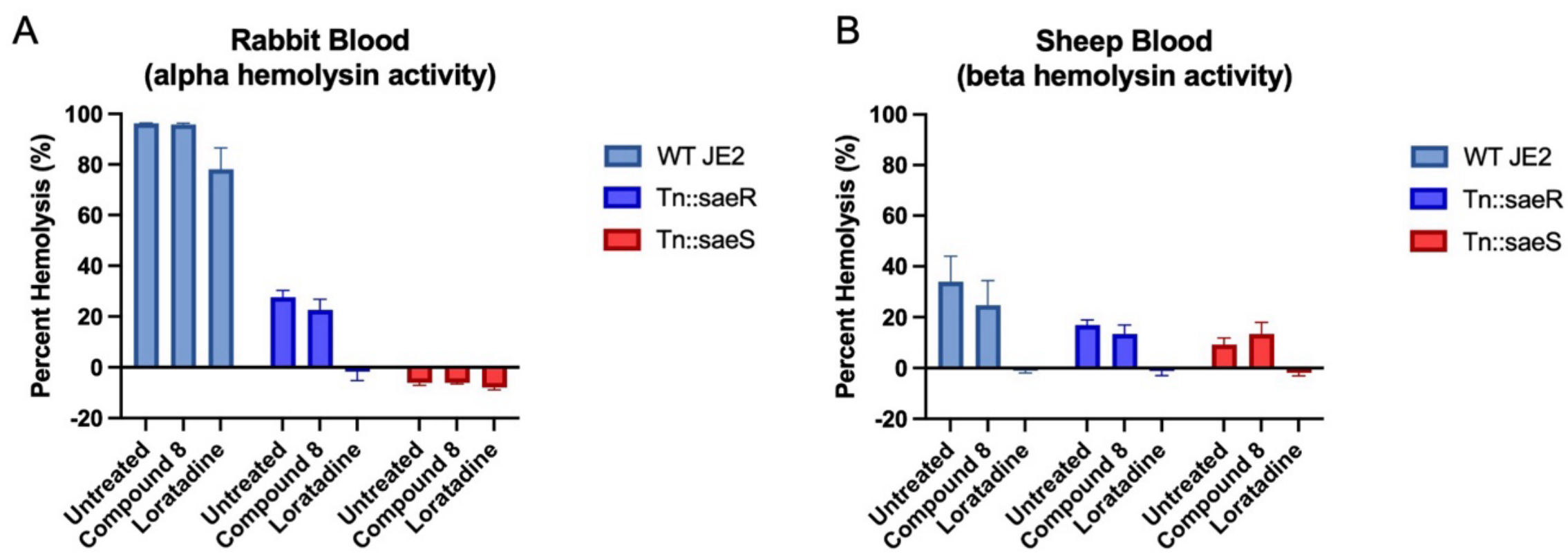

Figure S3: Quantitative hemolysis assays using (A) rabbit or (B) sheep blood reveal SaeRS is not required for loratadine's reduction in alpha or beta hemolytic activity in USA300 strain JE2. Compound 8 results are also included. In both panels, mean percent hemolysis is displayed from three biological replicates. Error bars represent the standard error of the mean. Two-way ANOVAs were used to assess variance between means of every sample. Details of this analysis are shared in Tables S1 for loratadine and S6 for compound 8.

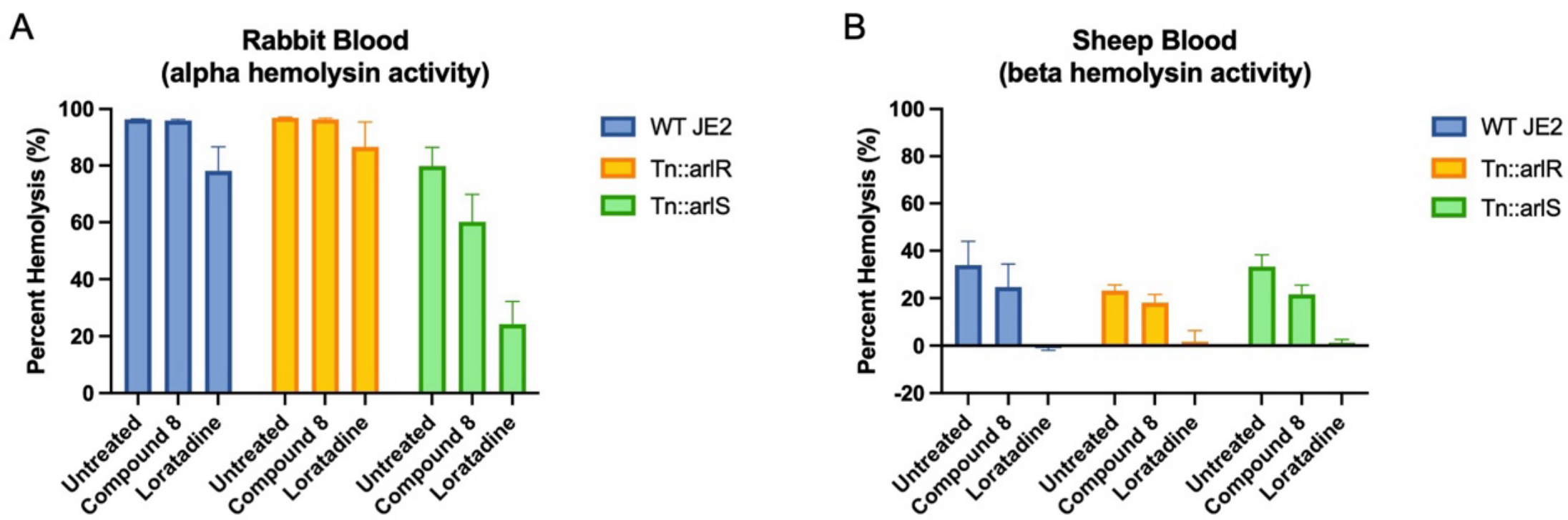

Figure S4: Quantitative hemolysis assays using (A) rabbit or (B) sheep blood reveal ArlS inhibiting loratadine's reduction of alpha hemolytic activity in USA300 strain JE2. Compound 8 results are also included. In both panels, mean percent hemolysis is displayed from three biological replicates. Error bars represent the standard error of the mean. Two-way ANOVAs were used to assess variance between means of every sample. Details of this analysis are shared in Tables S1 for loratadine and S6 for compound 8.

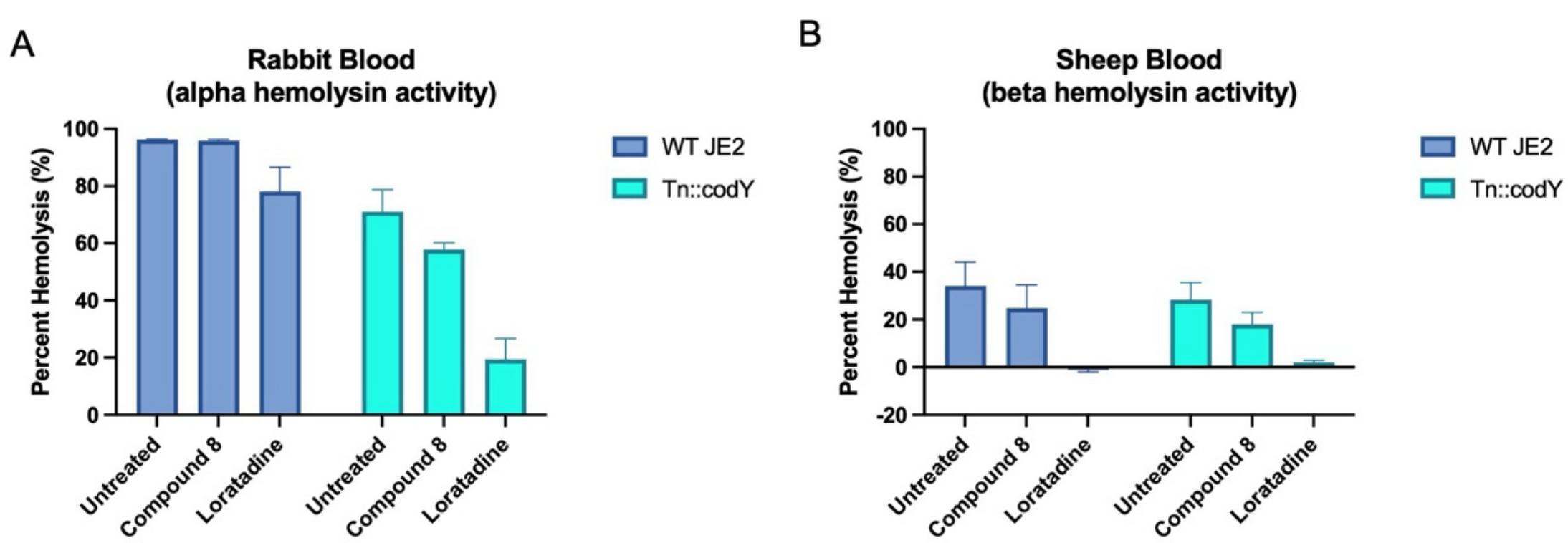

Figure S5: Quantitative hemolysis assays using (A) rabbit or (B) sheep blood reveal CodY inhibiting loratadine's reduction of alpha hemolytic activity in USA300 strain JE2. Compound 8 results are also included. In both panels, mean percent hemolysis is displayed from three biological replicates. Error bars represent the standard error of the mean. Two-way ANOVAs were used to assess variance between means of every sample. Details of this analysis are shared in Tables S1 for loratadine and S6 for compound 8.

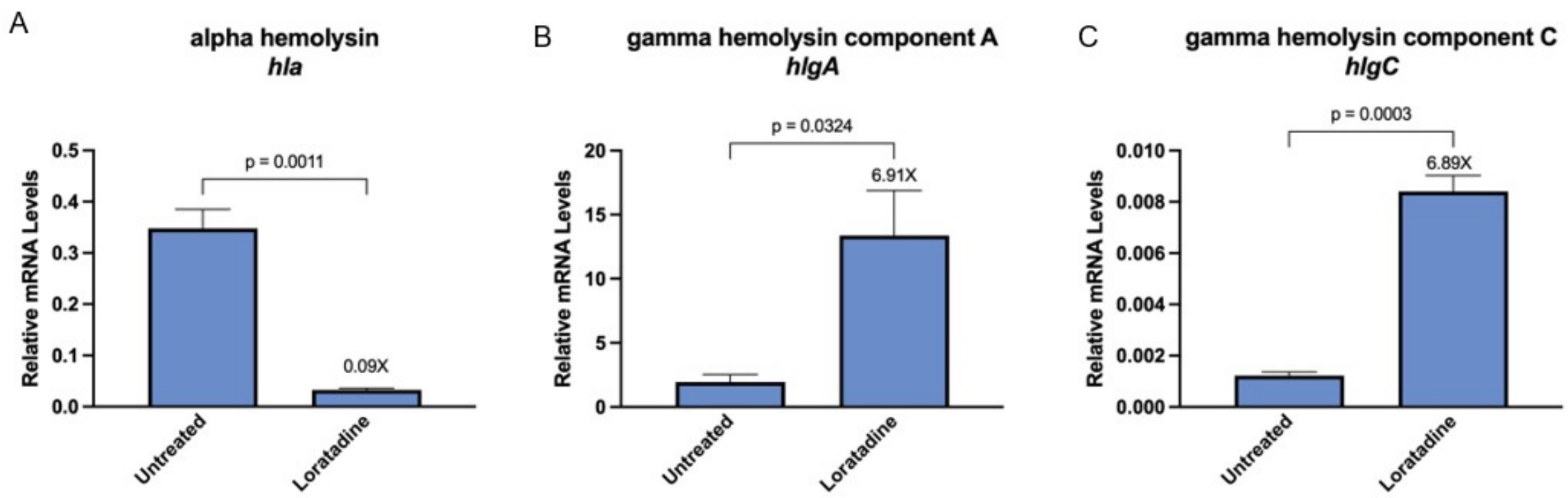

Figure S6: RT-qPCR validates multiple hemolysin genes changing with loratadine treatment. (A) *hla* (B) *hlgA* (C) *hlgC*. In all panels, mRNA levels are relative to a 16S reference gene. Error bars represent the standard error of the mean. Labels over treated samples indicate fold change from untreated controls. Unpaired student's t-tests were used to assess statistically significant differences. Resulting p values ≤0.05 are displayed.

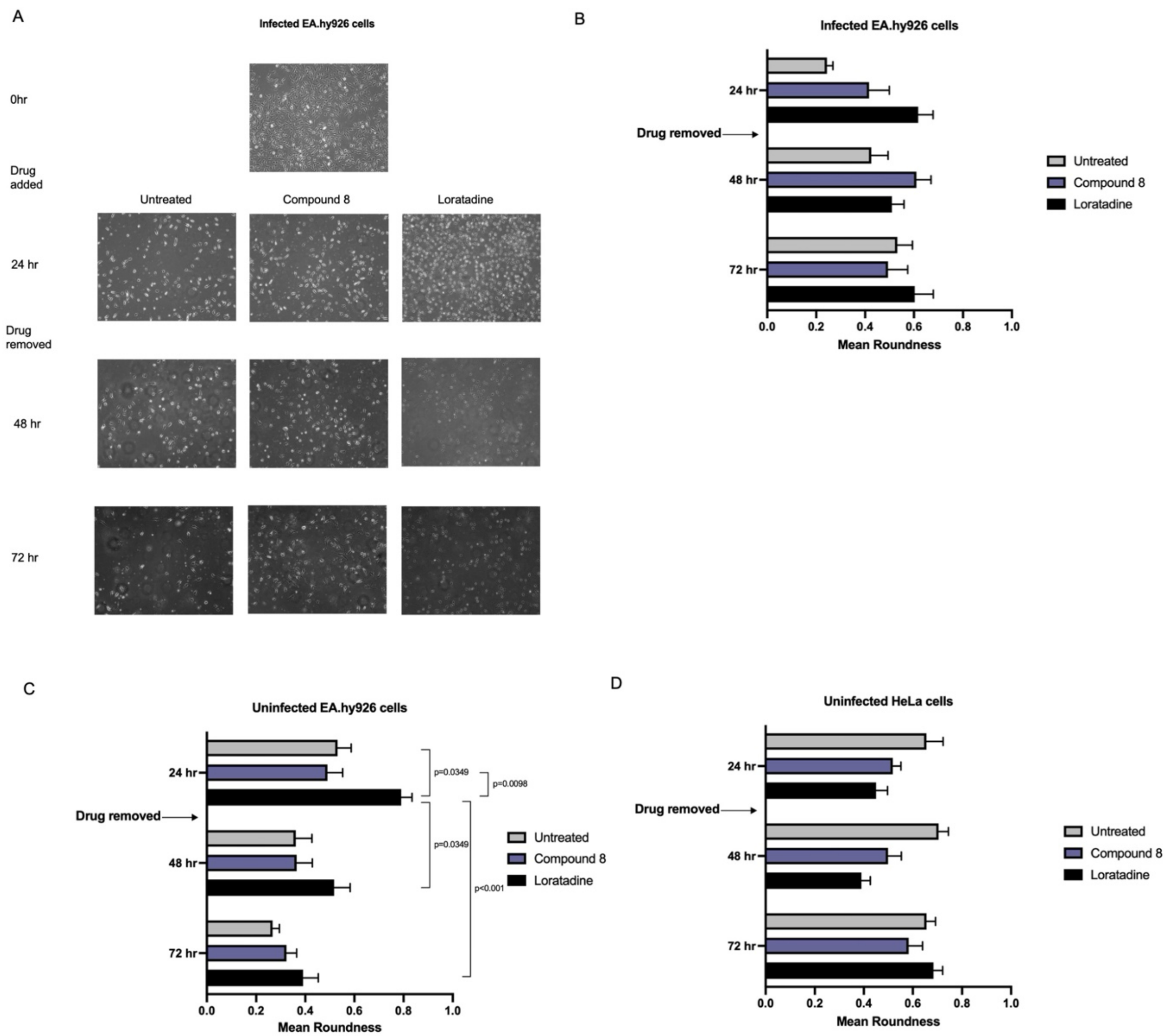

Figure S7: Loratadine-induced rounding of endothelial-like EA.hy926 cells is temporary. (A) Brightfield microscopy images of infected EA.hy926 cells over time. (B) Measurement of mean roundness of infected cells shown in panel (A). (C) Measurement of mean roundness of uninfected EA.hy926 cells. (D) Measurement of mean roundness of uninfected HeLa cells (epithelial). In all panels, mean roundness values can range from 0.0-1.0, with 1.0 being a perfect circle. Error bars represent the standard error of the mean. Adjusted p values ≤0.05 are displayed. Details of 2-way ANOVAs are found in Tables S38-40.

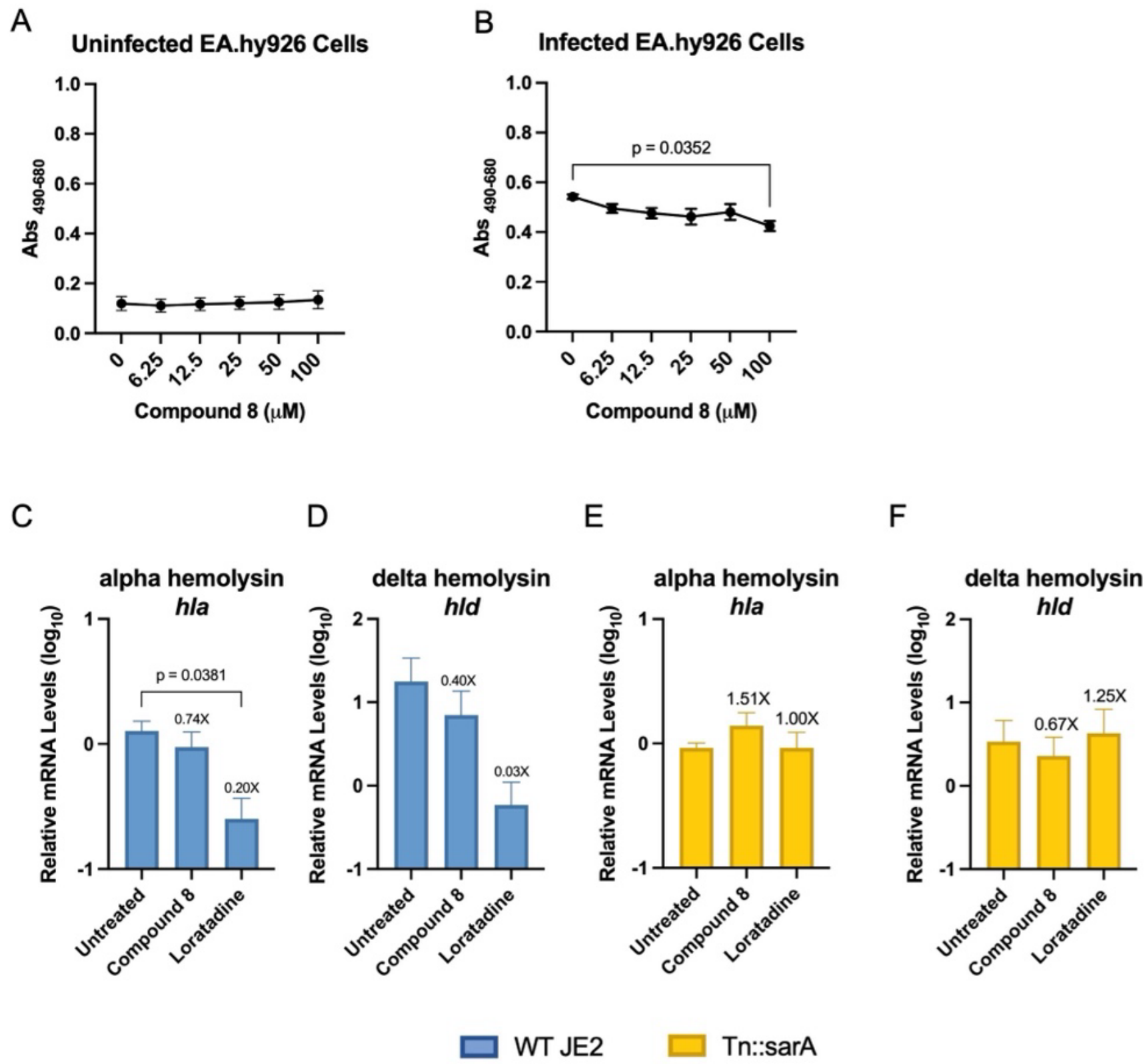

Figure S8: Human cell line infection model shows compound 8 does not induce cytotoxicity in a human cell line and multiple hemolysins' mRNA levels get suppressed. (A) Cytotoxicity results of compound 8 in EA.hy926 cells after 24 hrs (B) The same experiment as shown in (A), but in the presence of USA300 JE2 MRSA. Error bars represent the standard error of the mean. RT-qPCR results of (C) *hla* and (D) *hld* levels in internalized USA300 JE2 after 24 hrs of compound 8 treatment. RT-qPCR results of (E*) hla* and (F) *hld* in internalized USA300 JE2 Tn::sarA after 24 hrs of compound 8 treatment. In panels C-F, mRNA levels are relative to a 16S reference gene. Error bars represent the standard error of the mean. Labels over treated samples indicate fold change from untreated controls. Unpaired student's t-tests were used to assess statistically significant differences. Resulting p values ≤0.05 are displayed.

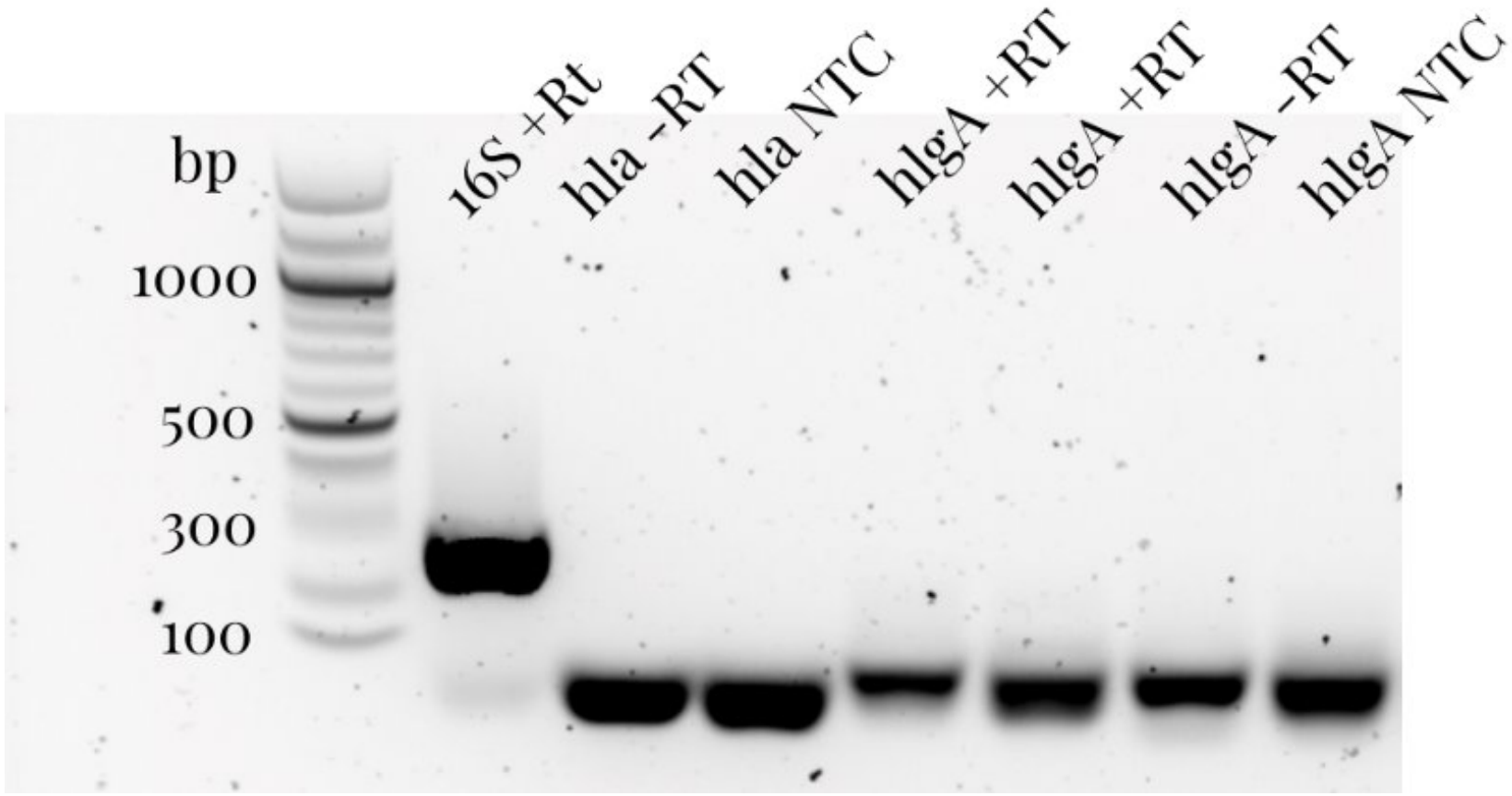

Figure S9: Agarose gel electrophoresis of RT-qPCR products. The presence of primer dimers only in hemolysin PCR reactions are verified for internalized USA300 JE2 Tn::stp1. A product of the expected size in 16S reference gene reactions is also verified. Experimental reactions that contained reverse transcriptase are labelled +RT, the controls for genomic DNA contamination are labeled –RT, and those that lacked template are labelled NTC for no template control.

References

1. Yarwood JM, McCormick JK, Paustian ML, Kapur V, Schlievert PM. 2002. Repression of the Staphylococcus aureus accessory gene regulator in serum and in vivo. J Bacteriol 184:1095-101.
2. Balogh H, Anthony AK, Stempel R, Vossen L, Federico VA, Valenzano GZ, Blackledge MS, Miller HB. 2024. Novel anti-virulence compounds disrupt exotoxin expression in MRSA. Microbiol Spectr 12:e0146424.
3. Viering B, Cunningham T, King A, Blackledge MS, Miller HB. 2022. Brominated carbazole with antibiotic adjuvant activity displays pleiotropic effects in MRSA's transcriptome. ACS Chem Biol 17:1239-1248.
4. Zheng W, Cai X, Xie M, Liang Y, Wang T, Li Z. 2016. Structure-Based Identification of a Potent Inhibitor Targeting Stp1-Mediated Virulence Regulation in Staphylococcus aureus. Cell Chem Biol 23:1002-13.
